# Giovanni Battista Grassi and Malaria

**DOI:** 10.3201/eid3204.AC3204

**Published:** 2026-04

**Authors:** Stefano Pozzi, Michele A. Riva

**Affiliations:** University of Milano–Bicocca School of Medicine and Surgery, Monza, Italy (S. Pozzi, M.A. Riva); Fondazione IRCCS San Gerardo dei Tintori, Monza (M.A. Riva)

**Keywords:** malaria, Giovanni Battista Grassi, Andrea Marrapodi, Gregorio Pampinella, Daniele Tozzi, art-science connection

**Figure 1:**
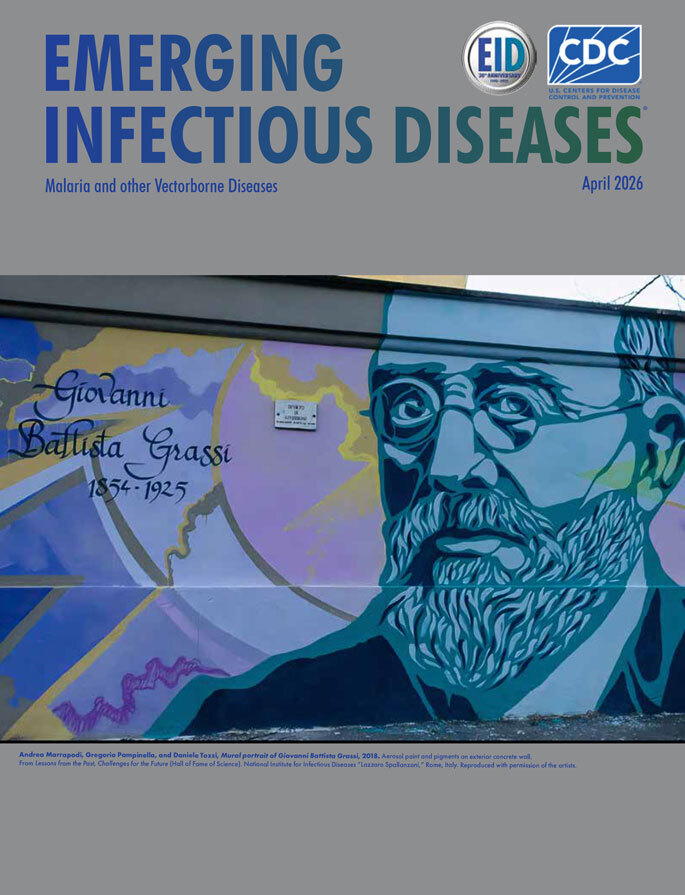
**Andrea Marrapodi, Gregorio Pampinella, and Daniele Tozzi, *Mural portrait of Giovanni Battista Grassi*, 2018.** Aerosol paint and pigments on exterior concrete wall. From *Lessons from the Past, Challenges for the Future* (Hall of Fame of Science). National Institute for Infectious Diseases “Lazzaro Spallanzani,” Rome, Italy. Reproduced with permission of the artists.

This month’s cover features a mural portrait of Giovanni Battista Grassi (1854–1925), created as part of the public art project *Lessons from the Past, Challenges for the Future* at the National Institute for Infectious Diseases “Lazzaro Spallanzani” in Rome, Italy (Figure). The installation, conceived as a “Hall of Fame of Science,” extends along 270 meters of the institute’s perimeter wall (≈810 m^2^) and presents 13 scientists who shaped the history of biomedical and infectious disease research. The mural was executed by 3 Italian artists—Andrea Marrapodi (KIV TNT), Gregorio Pampinella (Greg Jager), and Daniele Tozzi—working collaboratively through a structured division of roles: chromatic backgrounds, portraits, and calligraphic elements. Created using aerosol paints and pigments applied directly onto the exterior concrete wall, the work integrates the wall’s architectural seams into the composition. Grassi’s likeness appears to derive from historical photographic sources, reinterpreted through a contemporary graphic-muralist style. Curated by Graffiti Zero, the project was commissioned to mark the institute’s 80th anniversary and to symbolically link scientific heritage with present and future challenges.

**Figure 2:**
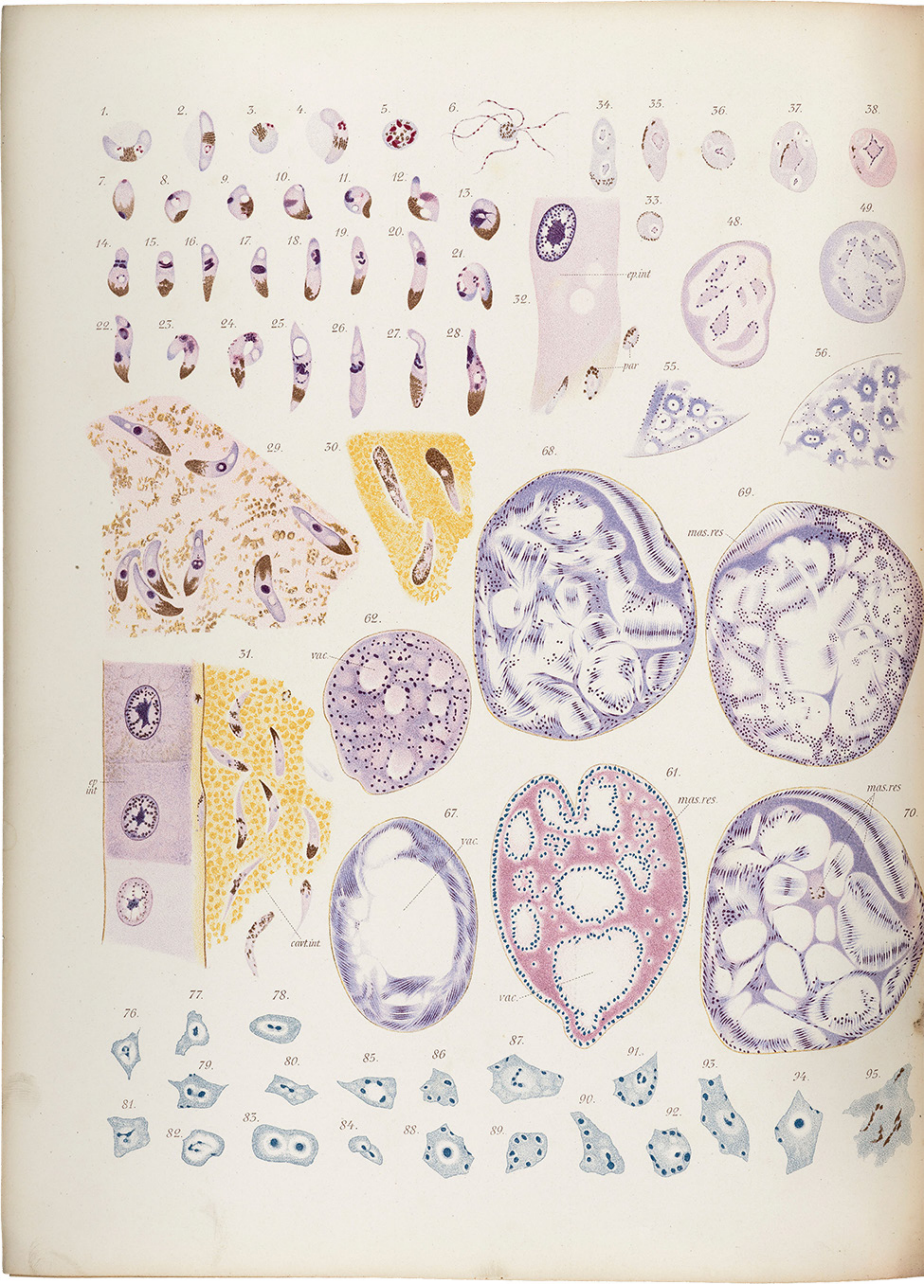
The life cycle of the malaria parasite, from Giovanni Battista Grassi’s *Studies of a Zoologist on Malaria*. Drawn with a camera lucida directly at the microscope, the figures capture with extraordinary precision the life cycle of the malaria parasite. Source: Wellcome Collection, London, UK (https://wellcomecollection.org/works/baxa2uau).

By situating Grassi among pioneers of microbiology and infectious disease research, the mural underscores the continuity of scientific inquiry and the enduring relevance of his contributions to the understanding of malaria. The recent centenary of the death of this physician, zoologist, botanist, and entomologist provides an opportunity to revisit his scientific career, which was marked by original insights and a multidisciplinary approach to infectious diseases. He investigated parasites such as *Ancylostoma duodenale* and *Hymenolepis nana*, defined the role of fleas as vectors of *Taenia cucumerina*, studied flagellates aiding wood digestion in termites, identified houseflies as disease carriers, and researched eels and *Phylloxera vastatrix* insects to protect vineyards. He discovered the arachnid *Koenenia mirabilis* and examined diseases with strong social and environmental determinants, including phosphorus necrosis and endemic goiter.

Yet it was malaria that became his defining challenge. In 1890, working with clinician Raimondo Feletti, he identified *Plasmodium vivax* parasites. Building on Alphonse Laveran’s hypothesis of mosquito involvement, Patrick Manson’s studies on filariasis, and Ronald Ross’s research on avian malaria, Grassi noted that malaria occurred only in certain mosquito-infested areas. This observation led him to propose that malaria was transmitted to humans by a specific group of mosquitoes. In 1898, through careful biogeographic study, he identified this group as belonging to the genus *Anopheles*.

That conclusion, reached with collaborators Giuseppe Bastianelli and Amico Bignami, was met with resistance. Robert Koch, visiting Rome that same year, rejected the *Anopheles* mosquito as a vector, citing its presence in malaria-free Grünewald near Berlin. Grassi persisted, and on November 1, 1898, he produced the first experimentally induced case of malaria in a healthy volunteer bitten by an *Anopheles claviger* mosquito, proving Koch wrong. Grassi emphasized the biologic complexity and specific ecology of *Anopheles* spp. mosquitoes, underlining Koch’s limited entomological expertise. In 1899, he demonstrated that infection in mosquitoes occurs only after feeding on infected humans, formulating what became known as Grassi’s Law: malaria = *Anopheles* + infected humans. Grassi urged the Italian Parliament to combine chemical protection with quinine and mechanical barriers—solid houses, metal screens—to prevent transmission, one of the earliest explicit proposals to use physical barriers against mosquitoborne infection. In direct relation to that last proposal, in 1899–1900, Grassi organized a large-scale experiment near Paestum, a malaria-endemic area. For several months, residents were monitored to assess whether metal nets installed on doors, windows, and chimneys could prevent malaria transmission. The results confirmed the accuracy of Grassi’s research: with few exceptions, all protected residents remained disease-free, whereas unprotected participants became infected.

The story of the discovery of the *Anopheles* mosquito as the vector of human malaria was marked by intense rivalry. The 1902 Nobel Prize in Physiology or Medicine, awarded to Ross, reflected a dispute that went beyond personal pride. Ross accused Italian scientists of following his lead, claiming that his description of a “gray-spotted mosquito with wings” had guided Grassi. In truth, the two men embodied opposing scientific philosophies: Ross relied on analogy and intuition from avian malaria; Grassi demanded systematic, comparative experiments in human malaria, warning that analogies between diseases with different vectors, parasites, and hosts were scientifically unsound. The resolution of the controversy was further complicated by the involvement of English physician Edmonston Charles, who visited Grassi’s laboratories in 1897–1898 and later reported his observations to Ross, and by the Swedish Royal Academy’s ill-judged choice of Koch as a neutral referee. Given Koch’s prior misjudgment on *An. claviger* mosquitos and his disagreements with Grassi, that decision only deepened tensions. Many historians now distinguish the achievements: Ross was the first to demonstrate transmission of avian malaria by mosquitoes, whereas Grassi was the first to identify and conclusively prove the vector of human malaria.

Grassi’s work extended far beyond this rivalry. In 1896, the Royal Society of London awarded him the Darwin Medal for his studies on termites and eels. He was elected to the Royal Academy of Sciences and appointed Senator of the Kingdom of Italy in 1908 for scientific merit. He continued working until his final days, revising a manuscript on *An. superpictus* mosquitoes before his death.

Grassi’s commitment to science is evident even in his meticulous drawings. At a time when photomicrography was not yet in widespread use, he recorded observations under the microscope by using a camera lucida, a device attached to the eyepiece that projected the image onto a surface for faithful reproduction. Plates such as those in *Studies of a Zoologist on Malaria* (Figure), produced more than a century ago, document the methods Grassi used to record microscopic observations. They illustrate an approach to scientific investigation based on careful observation, critical evaluation, and repeated verification.

Today, malaria continues to affect hundreds of millions of humans worldwide, despite decades of research, preventive programs, and pharmaceutical advances. The struggle against this ancient scourge illustrates the enduring relevance of Grassi’s approach: understanding disease requires careful observation, attention to ecologic and biologic complexity, and collaboration across disciplines.
